# Prognostic impact of p53, c-erbB-2 and epidermal growth factor receptor on head and neck carcinoma

**DOI:** 10.1590/S1516-31802004000600007

**Published:** 2004-11-04

**Authors:** Orlando Parise, Leda Viegas Carvalho, Roberto Elias Villela Miguel, Luiz Paulo Kowalski

**Keywords:** Squamous cell carcinoma, Genes p53, Genes c-erbB-2, Protein p53, Protein c-erbB-2, Carcinoma espinocelular, Genes p53, Genes c-erbB-2, Proteína p53, Proteína c-erbB-2

## Abstract

**CONTEXT:**

p53, c-erbB-2 and epidermal growth factor receptor (EGFR) are cancer-related proteins that are usually expressed in head and neck squamous cell carcinoma (SCC). Their prognostic value remains controversial.

**OBJECTIVE:**

To evaluate the prognostic impact of p53, c-erbB-2 and EGFR expression in head and neck SCC.

**TYPE OF STUDY:**

Prospective.

**SETTING:**

Head and Neck Surgery Department, Hospital AC Camargo, São Paulo.

**METHODS:**

Fifty-four patients were studied for p53, c-erbB-2 and EGFR expression in head and neck SCC and adjacent mucosa, via immunohistochemistry. These data were correlated with histoclinical data and survival.

**RESULTS:**

There was a direct association of p53 expression in SCC and mucosa (p = 0.001); loss of c-erbB-2 expression (-) from normal mucosa to SCC (p = 0.04); lower frequency of association of c-erbB-2 (+) with EGFR (-) in SCC (p = 0.02); and a direct association of EGFR (+) expression in SCC and mitotic index (p = 0.03). The 60-month actuarial survival rates for patients presenting lymph node metastasis were higher when there was no capsule rupture by SCC (48.3%; p = 0.02), no more than one positive lymph node (52.3%; p = 0.004) or clear surgical margins (47.0%; p = 0.01), in comparison with patients presenting capsule rupture (20.2%), two or more positive lymph nodes (18.7%) or compromised surgical margins (0.0%), respectively. Patients presenting SCC p53 (+) and EGFR (-) demonstrated greater survival (75.0%; p = 0.03) than for the remaining group (33.1%). Multivariate analysis confirmed the positive impact of p53 (+) and EGFR (-) on survival (p = 0.02).

**DISCUSSION:**

Associations were found for p53, c-erbB-2 and EGFR expression with histoclinical data and prognosis. Interestingly, these results suggest that loss of mucosal c-erbB-2 expression could be involved in SCC carcinogenesis; EGFR expression in SCC is related to tumor mitotic index; and presence of p53 with absence of EGFR expression in head and neck SCC may be a prognostic factor for survival.

**CONCLUSIONS:**

Further prospective studies should be conducted to confirm the influence of p53, c-erbB-2 and EGFR on histoclinical data and prognosis.

## RESUMO

**CONTEXTO:**

p53, c-erbB-2 e receptor epidermal do fator de crescimento (EGFR) são proteínas associadas ao câncer, geralmente expressas nos carcinomas espinocelulares (CEC) de cabeça e pescoço, porém seu valor prognóstico permanece controverso.

**OBJETIVO:**

Avaliar o impacto prognóstico da expressão de p53, c-erbB-2 and EGFR nos CECCP.

**TIPO DE ESTUDO:**

Prospectivo.

**LOCAL:**

Departamento de Cirurgia de Cabeça e Pescoço e Otorrinolaringologia do Hospital AC Camargo, São Paulo.

**MÉTODOS:**

A expressão de p53, c-erbB-2 and EGFR em CECCP e mucosas de 54 pacientes foram estudadas por imunoistoquímica e estes dados foram correlacionados com dados histoclínicos e sobrevida.

**RESULTADOS:**

Foram observadas: associação direta da expressão de p53 em CEC e mucosas (p = 0,001); perda da expressão de c-erbB-2 (-) da mucosa normal para o CEC (p = 0,04); menor freqüência da associação da expressão de c-erbB-2 positivo com EGFR negativo nos CEC (p = 0,02); e entre o EGFR positivo e o índice mitótico (p = 0,03). A sobrevida atuarial para 60 meses foi maior para os pacientes que apresentavam disseminação para gânglios no pescoço sem ruptura capsular (48.3% p = 0,02), até um gânglio positivo (52.3%, p = 0,004), e margens cirúrgicas livres (47.0%, p = 0,01), quando comparadas aos pacientes que apresentavam ruptura capsular nos gânglios do pescoço (20,2%), dois ou mais gânglios positivos (18,7%), e margens cirúrgicas comprometidas (0,0%), respectivamente. Pacientes apresentando CEC p53 positivo e EGFR negativo apresentaram uma sobrevida maior (75.0%, p = 0,03) quando comparados ao grupo de pacientes remanescentes (33,1%). A análise multivariada confirmou o impacto positivo de p53 positivo e EGFR negativo na sobrevida (p = 0,02).

**DISCUSSÃO:**

Este estudo encontrou associações da expressão de p53, c-erbB-2 e EGFR com dados histoclínicos e com o prognóstico. De especial interesse, os resultados sugerem que a perda da expressão mucosa de c-erbB-2 pode estar envolvida com a carcinogênese; que a expressão de EGFR está relacionada com o índice mitótico dos CEC e que a presença da expressão de p53 com perda da expressão de EGFR nos CEC pode ser um fator prognóstico.

**CONCLUSÕES:**

Estudos propectivos adicionais devem ser realizados para confirmar a influência de p53, c-erbB-2 e EGFR nos dados histoclínicos e na sobrevida.

## INTRODUCTION

Head and neck squamous cell carcinomas (SCC) are associated with smoking and alcohol consumption and are often preceded by preneoplastic lesions. In addition, they vary considerably in aggressiveness, metastatic potential and sensitivity to radiotherapy and chemotherapy. Histoclinical parameters alone do not consistently predict their potential aggressiveness and the outcome after standard treatment.

Probably the protein most studied in oncology over the last few years is p53. Many reviews have confirmed its crucial role in carcinogenesis as a tumor suppressor gene, but its value as a prognostic factor for head and neck squamous cell carcinomas remains controversial.^1-3^ It is generally accepted that p53 hyperexpression is the consequence of an increase in the half-life of the non-functional p53 protein. This is usually related to a mutation of the gene and is easily detectable via immunohistochemistry. Different mutations of p53 have been detected in the mucosa at sites that are distant from primary head and neck squamous cell carcinomas,^1^ thus suggesting that p53 mutations may be an early event in the carcinogenic process and, consequently, in multiple tumor genesis. This concept has been supported by the reporting of altered p53 expression in association with increased genetic instability in preneoplastic mucosa of the head and neck.^2^

c-erbB-2 is a so-called HER2-neu proto-oncogene and is a transmembrane protein with protein kinase activity. Most of the available information concerning c-erbB-2 expression has been obtained from studies of breast cancer, in which c-erbB-2 gene amplification or overexpression has been linked mainly to poor prognosis. c-erbB-2 expression has been detected in 40 to 60% of head and neck squamous cell carcinomas. With regard to preneoplastic lesions, c-erbB-2 expression has been observed in lichens but not in the corresponding head and neck squamous cell carcinomas arising from lichens, thus suggesting that the loss of c-erbB-2 expression is a carcinogenic molecular event.^4^ Paradoxically, it has been reported that, as the oral mucosa goes from normal to malignant phenotype, c-erbB-2 expression increases. In head and neck squamous cell carcinomas of the oral cavity, overexpression of the c-erbB-2 has been correlated with shorter overall survival.^5^

Epidermal growth factor receptor (EGFR) is highly similar to the c-erbB-2 protein in its intracytoplasmic domain. Both are members of the type 1 growth factor receptor family. EGFR has two main external ligands: epidermal growth factor and transforming α growth factor. Hyperexpression of EGFR is frequently observed in many human tumors and its blockage by monoclonal "antisense" antibodies can promote inhibition of the malignant phenotype.^6,7^ Despite the fact that the real role of EGFR in head and neck squamous cell carcinomas remains controversial, the co-expression of p53 and EGFR on bronchial epithelium seems to be related to SCC predisposition.^8^ Furthermore, a progressive increase has been noted in EGFR expression as the laryngeal epithelium changes from normal mucosa to hyperplastic mucosa and dysplastic mucosa.^9^ Among the erb family, EGFR appears to be the dominant component, controlling infiltration and the metastatic potential of head and neck squamous cell carcinomas.^10^

In order to evaluate molecular alterations that are potentially associated with aggressiveness of head and neck squamous cell carcinomas, we studied the prognostic impact of the immunohistochemical expression of p53, c-erbB-2 and EGFR on excised head and neck squamous cell carcinomas and adjacent normal mucosa.

## MATERIAL AND METHOD

### Clinical data and treatment protocol

The association between general clinical data, head and neck squamous cell carcinoma staging, histopathological findings and mitotic index were prospectively studied using the immunohistochemical expression of p53, c-erbB-2 and EGFR in head and neck squamous cell carcinomas and normal adjacent mucosa from patients who had not been treated previously. These patients were treated at Hospital AC Camargo (São Paulo, Brazil) between June 1995 and September 1996. All specimens were obtained in the operating room and immediately preserved in 10% buffered formalin (Carlo Erba) and formalin-acetic acid solution. The general guidelines for treatment consisted of surgery alone for initial clinical stages and surgery and radiotherapy for advanced lesions. Some cases with lymph node capsule rupture by tumor also underwent cisplatin-based chemotherapy concomitant with radiotherapy, as part of a clinical trial. Clinical data concerning tumor staging were prospectively collected.

### Immunohistochemistry

Immunohistochemical studies were performed on formalin-fixed paraffin-embedded tissue using the Avidin-Biotin-Peroxidase method.^11^ Four-micrometer sections were deparaffinized in xylene and rehydrated in descending grades of ethanol. Endogenous peroxidase activity was blocked with 3% hydrogen peroxide in methanol. To enhance the immunostaining of antibodies, a heat epitope-retrieval technique was utilized, with microwave pretreatment of the tissue sections. The primary antibodies used were "mouse anti-human p53 protein DO7" (1:25 dilution, Dako, Carpinteria, CA, USA) for p53 detection, "rabbit anti-human c-erbB-2 oncoprotein A485" (1:100 dilution, Dako, Carpinteria, CA, USA) for c-erbB-2 detection, and "mouse anti-human epidermal growth factor receptor H11" (1:100 dilution, Dako, Carpinteria, CA, USA) for EGFR detection. Immunoperoxidase staining was performed employing the "LSAB2 peroxidase kit" (Dako, Carpinteria, CA, USA) for p53 and c-erbB-2 reactions. EGFR was determined via the alkaline phosphatase method using the "en vision system alkaline phosphatase kit" (Dako, Carpinteria, CA, USA). Diaminobenzidine was used as the chromogen for the peroxidase reaction and "fast red" was used for the alkaline phosphatase reaction. Positive and negative controls were used. The results were analyzed qualitatively, and samples with 10% or more of the cells stained were considered positive, i.e. nuclear staining for p53 expression and membrane staining for c-erbB-2 and EGFR.

### Statistical analysis

Fisher exact test and the chi-squared test were used to compare differences in p53, c-erbB-2 and EGFR expression related to clinical and histological data. The actuarial survival was obtained by the Kaplan-Meier method and survival differences between groups were evaluated by the Mantel-Cox method. The Cox proportional hazards regression analysis was used in multivariate survival analysis. Differences with p ≤ 0.05 were considered significant.

## RESULTS

The mean age of the patients was 58.6 years (range: 33 to 88), with 44 males and 10 females. The tumor sites were distributed as follows: mouth in 25 patients (46.3%); oropharynx in 8 (14.8%); larynx in 13 (24.1%); and hypopharynx in 8 (14.8 %). Staging (based on TNM, tumour node metastasis classification, all patients were M0) included: stage II 10 patients; stage III 17; and stage IV 37. The following treatment was administered: combination surgery-radiotherapy 31 (57.3%); surgery alone 20 (37.0%); and combination surgery-radiotherapy-chemotherapy 3 (5.7%). Fifty-three out of 54 patients were submitted to a neck dissection with 25 N- and 28 N+ and, among them, 17 presented capsule rupture by SCC (N+R+). The surgical margins were considered clear for 44 patients (81.4%), close for 5 (9.3%), and positive for 5 (9.3%). With regard to SCC differentiation, 10 patients (18.5%) were poorly differentiated, 28 (51.9%) moderately differentiated and 16 (29.6%) well differentiated. Twenty-two patients (40.8%) had a mitotic index of less than 10 mitoses; 14 (33.3%) had a mitotic index of 10 to 20 mitoses; and 18 (25.9%) presented a mitotic index of more than 20 mitoses.

### Expression of p53, c-erbB-2, and EGFR

Fifty-four SCC samples were analyzed for p53 and c-erbB-2, and 53 SCC samples for EGFR. Thirty-two 32 adjacent mucosa samples were analyzed for p53, 33 adjacent mucosa samples for c-erbB-2 and 31 adjacent mucosa samples for EGFR.

The p53 protein was detected in 29/54 SCC (53.7%) and in 12/32 (37.5%) mucosa samples analyzed. A significant direct association between p53 in SCC and corresponding mucosa was detected (p < 0.001).

c-erbB-2 was detected in 21/54 (38.9%) SCC and in 20/33 (60.6%) mucosa samples analyzed. Significantly less frequent presence of c-erbB-2 in SCC, in association with absence of c-erbB-2 in the corresponding mucosa (p < 0.04), was observed .

EGFR was observed in 20/53 (37.7%) SCC and in 16/31 (51.6%) mucosa samples analyzed.

The paired analysis of p53, c-erbB-2 and EGFR expression in mucosa and SCC from the same patient is illustrated in Table 1. All possible associations between p53, c-erbB-2 and EGFR expression in mucosa versus mucosa, mucosa versus SCC, and SCC versus SCC were verified. Significantly less frequent presence of c-erbB-2 in SCC, in association with absence of EGFR in SCC, was found (p < 0.02, Table 2).

**Table 1:** p53, c-erbB-2 and epidermal growth factor receptor (EGFR) expression in head and neck squamous cell carcinomas (SCC) and corresponding mucosa (paired)

Mucosa	Head and neck SCC			
	**p53- n** (%)	**p53+ n** (%)		**Total n** (%)
p53 -	16 (50.0)	4 (12.5)		20 (62.5)
p53 +	1 (3.1)	11 (34.4)		12 (37.5)
N/A	7	15		22
**Total**	**24**	**30**		**54**
	**c-erbB-2-n** (%)	**c-erbB-2+n** (%)		**Total n** (%)
c-erbB-2 -	12 (36.4)	1 (3.0)		13 (39.4)
c-erbB-2 +	12 (36.4)	8 (24.2)		20 (60.6)
N/A	9	12		21
**Total**	**33**	**21**		**54**
	**EGFR-n** (%)	**EGFR+n** (%)	**N/A**	**Total n** (%)
EGFR -	8 (26.7)	7 (23.3)	0	15
EGFR +	4 (13.3)	11 (36.7)	1	16
N/A	8	15	0	23
**Total**	**20**	**33**	**1**	**54**

*p53- = p53 not detected; p53+ = p53 detected; c-erbB-2- = c-erbB-2 not detected; c-erbB-2 + = c-erbB-2 detected; EGFR- = EGFR not detected; EGFR+ = EGFR detected. N/A = not applicable, considering that 22 patients had no evaluation of p53 in mucosa to compare with the corresponding SCC; 21 patients had no evaluation of c-erbB-2 in mucosa to compare with the corresponding SCC; 23 patients had no evaluation of EGFR in mucosa to compare with the corresponding SCC; and one patient had no evaluation of EGFR in SCC to compare with the corresponding mucosa.*

**Table 2:** Detection of c-erbB-2 and epidermal growth factor receptor (EGFR) co-expression in head and neck squamous cell carcinomas (SCC) (paired)

	Head and neck SCC		
	**c-erbB-2-n** (%)	**c-erbB-2+n** (%)	**Total n** (%)
EGFR -	16 (30.2)	4 (7.5)	20 (37.7)
EGFR +	16 (30.2)	17 (32.1)	33 (62.3)
N/A	1	0	1
**Total**	**33**	**21**	**54**

*c-erbB-2 - = c-erbB-2 not detected; EGFR - = EGFR not detected; c-erbB-2 + = c-erbB-2 detected; EGFR + = EGFR detected; N/A = not applicable, since one patient had no evaluation of EGFR in SCC.*

A tendency was also noted towards more frequent presence of p53 in oral SCC than in the hypopharynx (p = 0.07). Furthermore, a significant direct association between the presence of EGFR expression in SCC and the mitotic index was observed (p = 0.03, Table 3).

**Table 3:** Epidermal growth factor receptor (EGFR) expression in head and neck squamous cell carcinomas (SCC) in relation to mitotic index

Mitotic index	Head and neck SCC		
	**EGFR-n** (%)	**EGFR+ n** (%)	**Total n** (%)
< 10	12 (22.6)	9 (17.0)	21 (39.6)
≥ 10 < 20	6 (11.3)	8 (15.1)	14 (26.4)
≥ 20	2 (3.8)	16 (30.2)	18 (34.0)
**Total**	**20**	**33**	**53** *

*EGFR - = EGFR not detected; EGFR + = EGFR detected;*

* *among the 54 patients, one patient had no evaluation of EGFR in SCC to compare with the mitotic index*.

### Survival Analysis

With regard to outcome, the follow-up varied from 10 days to 73.4 months. During this period, 33 deaths occurred and 5 patients were lost to follow-up. The overall survival at 36 and 60 months was 48.9 % and 39.4%, respectively. Table 4 shows all factors with a significant impact on survival: patients with absence of capsule rupture of metastatic lymph node, no more than one lymph node histologically positive or clear surgical margins had a better prognosis than for patients presenting lymph nodes with capsule rupture by SCC, two or more lymph nodes histologically positive or involved surgical margins, respectively. The impact on patient survival of clinical N0 and/or histological N- presentation that was observed at 36 months was not maintained at 60 months (Table 4).

**Table 4:** Significant variables relating to actuarial survival at 36 and/or 60 months

Variable	Group	36 months % survival	p	60 months % survival	p
Clinical node	N0	57.0	0.04	46.1	NS
	N1-3	40.0		32.0	
Neck histology	N-	62.8	0.05	47.8	NS
	N+	36.7		32.2	
Node capsule rupture	Remaining	58.6	0.008	48.3	0.02
	N+R+	26.9		20.2	
Number of positive neck nodes	Up to 1	63.6	0.004	52.3	0.004
	2 or more	25.0		18.7	
Surgical margins	Clear	55.8	NS	47.0	0.01
	Compromised	20.0		0.0	
Expression of p53 and EGFR in head and neck SCC	P53+ EGFR-Remaining	87.5 41.6	0.04	75.0 33.1	0.03

With regard to the impact on survival of p53, c-erbB-2 and EGFR expression, patients presenting head and neck squamous cell carcinomas with p53 and absence of EGFR expression had a better survival rate (75.0%) than for the remaining group (33.1%). In order to verify a possible distribution bias among the clinical prognostic variables that could influence this result, we compared the primary site of the two groups, T (TNM), nodal histology and mitotic index. This demonstrated that there was no significant difference between the two groups. Multivariate analysis via the Cox proportional hazards regression, considering capsule rupture, number of positive lymph nodes, surgical margin status and p53 and EGFR expression in SCC, showed a tendency towards negative impact on survival when the surgical margins were compromised (p = 0.055; ExpB 2.34) and a significant positive impact on survival when the SCC had p53+ and EGFR- (p = 0.022; ExpB 0.19).

## DISCUSSION

This study found significant associations for p53 and c-erbB-2 expression in head and neck squamous cell carcinomas that were paired with the corresponding mucosa from the same patient. With regard to p53 expression, a direct association was observed between p53 in SCC and the corresponding mucosa. It can be speculated whether this in fact means a compromised margin from the molecular point-of-view or whether it reflects molecular alterations of the field of cancerization.^1^ This group with p53 expression did not display any increase in local recurrence, but an association between p53 detected in the surgical margin and local recurrence was previously reported by Ball et al.^12^ With regard to c-erbB-2 analysis, the significant reduction in the expected frequency of c-erbB-2 presence in head and neck squamous cell carcinomas and the absence of c-erbB-2 in the corresponding mucosa may be interpreted, from a biological point-of-view, as the normal mucosa presenting the c-erbB-2 expression and then losing it during cancer progression. A similar finding was reported by Kilpi et al., who found that lichen keratocytes lose their c-erbB-2 expression during cancer progression.^4^ In apparent contradiction, Wilkman et al.^13^ reported an increase in c-erbB-2 expression during the sequence from normal mucosa to hyperkeratosis and to dysplasia and head and neck squamous cell carcinoma. The problem with the latter study is that samples representing different stages of carcinogenesis were obtained from different patients. If it were confirmed that loss of c-erbB-2 expression is actually a molecular step that is required for normal mucosa to become SCC, c-erbB-2 expression could be used as a marker of cancer progression or be used to stratify clinical trials of preneoplastic lesions.

The paired analysis of p53, c-erbB-2 and EGFR expression in mucosa and SCC from the same patient showed significantly less frequent expression of c-erbB-2 in SCC in association with absence of EGFR in SCC (p < 0.02, Table 2). This could be explained by taking the hypothesis that downregulation of c-erbB-2 through EGFR expression is taking place. Inverse expression of EGFR and c-erbB-2 in kidney carcinomas has been reported.^14^ *In vitro*, TGF-α has a stimulating effect on c-erbB-2 and not on EGFR, thus suggesting a distinct modulatory effect.^15^ It has also been reported that a reduction in TGF-α concentration does not alter the growth rate of the normal pharyngeal epithelium, but reduces the growth rate of the SCC.^16^ We can hypothesize that, during carcinogenesis, at least part of the selective advantage of the SCC clone is obtained through reduction in c-erbB-2 expression and increased activity of EGFR expression.

A tendency was also noted towards more frequent presence of p53 in oral SCC than in the hypopharynx (p = 0.07). This may be interesting, since it seems that the prognostic value of p53 expression varies according to the primary tumor site. In hypopharyngeal SCC, the presence of p53 has been associated with good prognosis.^17^

A significant direct association between the presence of EGFR in SCC and the mitotic index was observed. This reinforces the idea that it might be clinically possible to inhibit SCC growth by blockage of EGFR, using monoclonal antibodies^18,19^ or antisense oligonucleotides.^7^ This latter strategy has shown selective reduction of SCC proliferation without changes in normal mucosa, thereby suggesting that it promotes antineoplastic activity with low toxicity to normal tissue.^20^

With regard to survival data, univariate analysis confirmed the well-established association of neck node metastasis with poor prognosis. In the present study, patients with lymph nodes presenting capsule rupture or two or more positive lymph nodes had a markedly lower survival rate than for those presenting lymph nodes without capsule rupture by SCC or no more than one positive lymph node. The impact on survival of clinical N1-3 and histological N+ that was observed at 36 months was not observed at 60 months, probably due to the curves crossing. The present study also suggests that presence of p53 and absence of EGFR in SCC might identify a subset of head and neck squamous cell carcinoma patients with good prognosis: this association was statistically significant in univariate and multivariate analyses. This leads to some hypotheses. We compared the distribution of tumor site, clinical T, histological N and mitotic index for two groups of head and neck squamous cell carcinomas (those with p53+ and EGFR-, versus the remaining patients) and no distribution bias was detected. In vitro, it has been demonstrated that EGFR hyperexpression downmodulates to a normal level when wild p53 protein is restored.^21^ It has also been reported that p53 hyperexpression is a good prognostic factor for tongue base^22^ and laryngeal^23^ carcinomas. The prognostic value of EGFR was reviewed recently, showing that EGFR expression in head and neck squamous cell carcinomas is associated with lower recurrence-free or overall survival.^24^ The prognostic value of p53 and EGFR expression and co-expression in head and neck squamous cell carcinomas merits further investigation.

## CONCLUSIONS

The data from this study suggest that loss of mucosal c-erbB-2 expression could be involved in squamous cell carcinogenesis; EGFR expression in head and neck squamous cell carcinomas is related to tumor mitotic index; and presence of p53 and absence of EGFR expression in SCC may be a prognostic factor for survival. Better biologically guided tumor characterization is fundamental for individualizing treatment for each patient, thereby possibly resulting in better control of head and neck squamous cell carcinomas.
